# Baseline Clinical Factors Associated with Cessation of Growth Hormone Therapy in Patients with Severe Growth Hormone Deficiency - Real World Evidence

**DOI:** 10.5812/ijem-147825

**Published:** 2024-10-27

**Authors:** Nageswary Nadarajah, Emmanuel Ssemmondo, Shani Brooks, Remi Akinyombo, Kazeem Adeleke, Harshal Deshmukh, Thozhukat Sathyapalan

**Affiliations:** 1 Allam Diabetes Centre, Hull University Teaching Hospitals NHS Foundation Trust, Hull, UK; 2 Academic Diabetes, Endocrinology and Metabolism, Allam Diabetes Centre, University of Hull, Hull, UK; 3 University of Cambridge, Cambridge, UK

**Keywords:** Growth Hormone, Clinical Factors, Therapy Cessation

## Abstract

**Background:**

Growth hormone replacement is indicated in adults with severe growth hormone (GH) deficiency, adult growth hormone deficiency assessment (AGHDA) score of at least 11 and are receiving treatment for other pituitary hormone deficiencies. There are no data looking at the cessation of GH replacement in adult patients with severe GH deficiency and the factors that predict the likelihood of patients continuing or stopping growth hormone replacement.

**Methods:**

We audited patients on the GH register between January 2006 and January 2023 in Hull University Teaching Hospitals NHS foundation Trust, a UK tertiary hospital. Baseline characteristics, the cause of GH deficiency, AGHDA score at diagnosis and the reason for stopping GH were collected. Proportions were compared between patients adhering to GH replacement and those who had ceased it. Logistic regression analysis was used to identify factors independently associated with cessation of GH.

**Results:**

The study comprised 141 adult patients with a mean age of 52 years, of which 75 (53%) were female. 54 (38%) individuals had discontinued GH replacement therapy.

Predominant reasons for discontinuation were lack of therapeutic benefit (46%) and a change in clinical indication (26%). Among patients who discontinued GH therapy, the most frequent cause of GH deficiency was idiopathic (57%), while for those on GH replacement, pituitary surgery was the leading cause of GH deficiency (53%). Logistic regression analysis showed no baseline factor was statistically significantly associated with GH cessation, except female gender which had a borderline significance (P = 0.05).

**Conclusions:**

In this real-world investigation of patients with severe GH deficiency, over two in five individuals who discontinued GH therapy cited the absence of perceived benefits. We show a borderline association of female gender with GH cessation and large population-based studies will be needed to investigate this and other causes of GH cessation.

## 1. Background

Growth hormone (GH) is an anabolic hormone secreted episodically from somatotroph cells in the anterior pituitary gland (1). It is regulated by three hypothalamic neuroendocrine hormones: Growth hormone-releasing hormone (GHRH), somatostatin (SST), and ghrelin (1-3). GH exerts its effects directly and indirectly. The direct effects of GH include its binding to target cells to stimulate a response. The indirect effects occur through insulin-like growth factor-1 (IGF-1), secreted by hepatocytes in response to GH stimulation (4, 5). Both GH and IGF-1 constitute the somatotropic axis responsible for the regulation of metabolism and physiological processes in the body (6). GH affects multiple organs and systems including muscle mass and bone mineral content among others (1, 7, 8).

 Adulthood growth hormone deficiency (GHD) may occur because of childhood-onset GHD or later in life (9). Growth hormone deficiency in children is commonly idiopathic. It often presents with short stature and low growth velocity for age (1). Organic causes of GHD in children include genetic mutations (GH-1, POU1F1, and PROP 1), pituitary stalk agenesis and septo-optic dysplasia (1, 9-12). Adult-onset GHD is frequently caused by pituitary adenoma and/or treatment with surgery or radiotherapy. Rare causes of adult GHD include craniopharyngioma, Sheehan’s syndrome, traumatic brain injuries and lymphocytic hypophysitis (1, 9, 10, 13, 14). 

Signs and symptoms of adult GHD manifest with changes in body composition and metabolism, reduced exercise capacity and quality of life. These include depression, reduced energy levels, increased anxiety, and decreased muscle mass (10, 15, 16). Diagnosis of adult-onset GHD can be challenging due to a lack of pathognomonic features (4). Growth hormone stimulation tests may help diagnose patients at risk of developing GHD (17). Severe GHD is established when a peak GH response of < 9 mU/litre (3 ng/mL) is observed during an insulin tolerance test (ITT) or another validated equivalent test [18]. If ITT is contraindicated (for example due to epilepsy or ischaemic heart disease), assessment can be done with either arginine, glucagon, levodopa, growth hormone-releasing hormone (GHRH) or clonidine stimulation test (1, 18).

 The treatment of adult GHD with recombinant human growth hormone (rhGH) is commenced in patients with severe GH deficiency, a ‘Quality of life assessment of growth hormone deficiency in adults’ (QoL-AGHDA) score of at least 11 and are receiving treatment for other pituitary hormone deficiencies. It is administered by a daily subcutaneous injection with some preparations requiring refrigeration (18, 19). Treatment is titrated with monthly assessments of serum IGF-1 levels for the first 2 - 3 months, until a maintenance dose is attained (18). Growth hormone replacement has shown beneficial effects on bone turnover, cardiovascular health, and quality of life (QoL) (7, 8, 13). As per National Institute for Health and Care Excellence (NICE) guidelines published in 2003, QoL status for patients receiving GH replacement should be re-assessed 9 months after commencing treatment and should be discontinued if the improvement is less than 7 points. Patients however, who were already on GH replacement by the time the guideline was published, were allowed to continue the treatment unless it was decided by the clinician and the patient that it was appropriate to stop (18).

The decision to continue GH replacement in adults lies solely in the improvement in the quality of life associated with GH replacement. There is however evidence to suggest that GH treatment interruption in under treated GHD patients may result in increased body fat and worsening lipid metabolism with higher levels of total cholesterol (20-22). It may also alter glucose metabolism by increasing fasting blood glucose levels (20). Conversely, prolonged GH treatment may be associated with fractures, adverse cardiovascular health, and cancer although the evidence from long-term clinical outcome studies is still deficient (23).

## 2. Objectives

There is no real-world data looking at cessation of GH replacement in patients diagnosed with severe growth hormone deficiency. We therefore sought to describe the clinical characteristics of patients who stop GH replacement, highlighting the common reasons for stopping and in addition, identify the factors that may predict which patients with GH deficiency are likely to stop GH replacement. 

## 3. Methods

This retrospective audit was done in Hull University Teaching Hospitals NHS Foundation Trust, a UK tertiary hospital. Electronic patient records of all individuals on the Growth Hormone register between Jan 2006 and Jan 2023 were reviewed. Anonymized details of each patient were entered into the growth hormone audit database. Data were collected on patients’ demographics, cause of growth hormone deficiency, date GH was started, date GH was stopped (as appropriate) and reason for stopping GH, AGHDA score prior to starting growth hormone (if available). 

**Figure 1.**
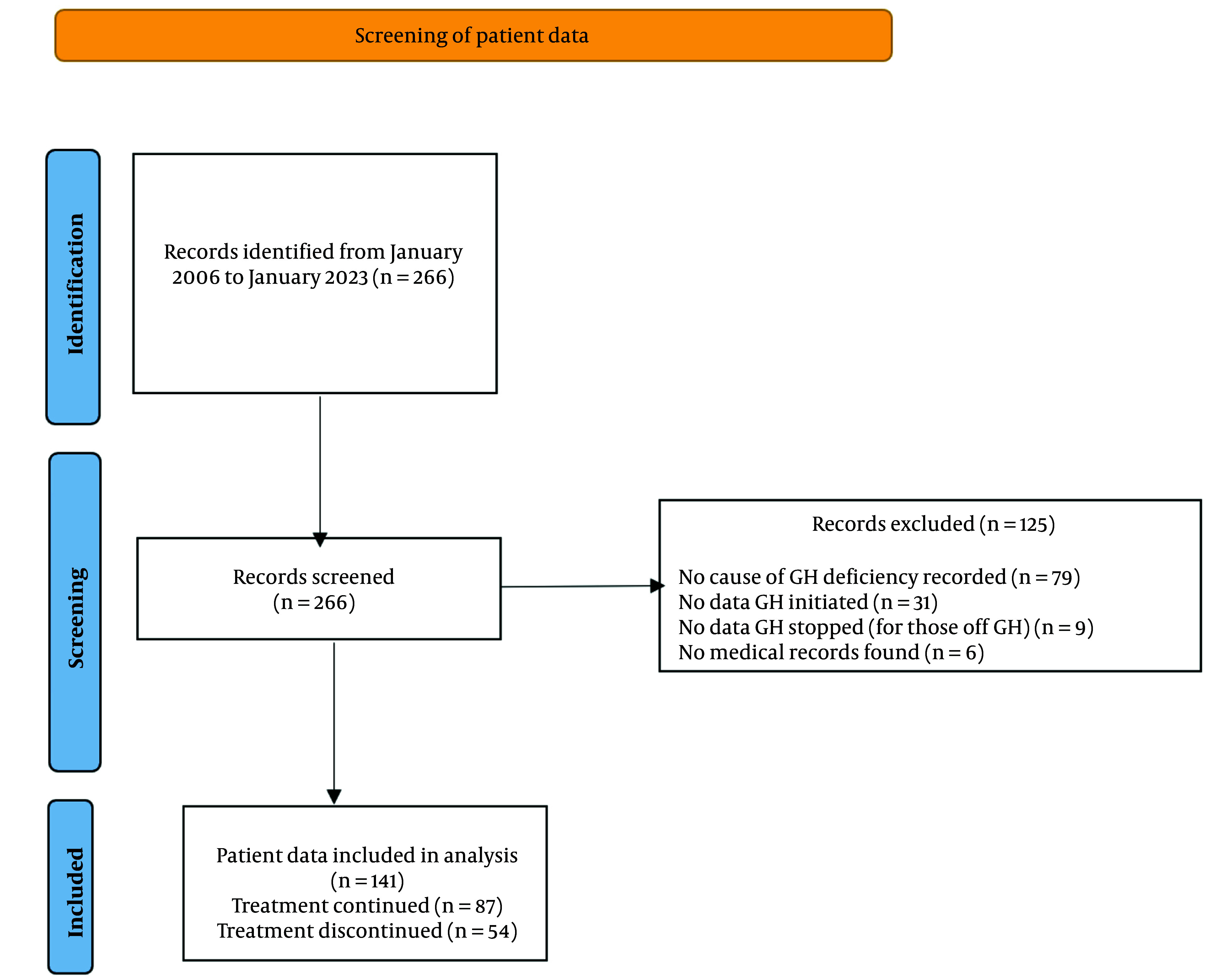
Flowchart diagram of patient data selection process

Two hundred and sixty-six patients were on the GH register. We, however, excluded one hundred and twenty-five patients. Of these, seventy-nine patients lacked adequate information regarding the cause of GH deficiency. In addition, thirty-one patients had no date GH was initiated, nine patients were off GH, but no stop date was available while six patients had no records on the hospital system (Figure 1). The remaining patients were divided into 2 groups; those on GH replacement (‘GH continued’, 87 patients) and those who were off GH (‘GH stopped’, 54 patients. For patients who underwent a dynamic test for diagnosis, severe GH deficiency was defined as a peak GH level of < 9 mU/litre (3 ng/mL) following the stimulation test. For patients who had a history of organic hypothalamic-pituitary disease and 3 or more hormone deficiencies, severe GH deficiency was diagnosed based on low serum IGF-1 levels (24).

### 3.1. Ethical Considerations

The audit was approved by the Hull University Teaching Hospital NHS Trust’s Audit and Clinical effectiveness team. There are guidelines followed with data collected anonymously at the point of collection and entry into the database.

### 3.2. Statistical Methods

Results are described as frequencies, mean and standard deviation as appropriate. Proportions were compared between patients continuing GH replacement (‘GH continued’ group), and those who were off GH (‘GH stopped’ group) using Chi-square test. To identify factors associated with cessation of GH replacement, cessation of GH replacement was modelled as a dependent variable (outcome) in a logistic regression model with age, gender, AGHDA score as independent predictors. Including the cause of GH deficiency in the model was producing unrealistic estimates because of presence of zeros in some of the groups, therefore was not included in the final model. All statistical analysis was done using R statistical software (v4.1.2; R Core Team 2021).

## 4. Results

One hundred and forty-one patients were included in the analysis. Of these, 87 were on GH replacement (‘GH continued’) while 54 (38%) were off GH replacement (‘GH stopped’). The ‘GH continued’ group had a mean age of 54 years while that of the ‘GH stopped’ group was 49 years. Overall, 66 (46.8%) of the patients were male. The ‘GH stopped’ group had a statistically significant higher proportion of females compared to the ‘GH continued’ group (65% Vs 46%) (P = 0.045). As shown in Table 1, most individuals in both groups were diagnosed with GHD using dynamic tests (GH continued group: 96.6%, GH stopped group: 94.4%) while diagnosis of the rest was based on low IGF-1 levels. 

**Table 1.** Baseline Characteristic of Study Participants ^a^

Variables	GH Continued (N = 87)	GH Stopped (N = 54)	P-Value
**Gender**			0.045
Male	47 (54)	19 (35)	
Female	40 (46)	35 (65)	
**Age (y)**	54.1 ± 16.7	48.9 ± 16.1	0.067
**Test used for diagnosis**			0.675
Dynamic test	84 (96.6)	51 (94.4)	
Low IGF-1 levels	3 (3.4)	3 (5.6)	
**Cause of GH deficiency**			< 0.001
Congenital	0 (0)	5 (9)	
Empty sella	0 (0)	3 (6)	
Hypophysitis	0 (0)	15 (28)	
Idiopathic	21 (24)	31 (57)	
Pituitary surgery	46 (53)	0 (0)	
Trauma	12 (14)	0 (0)	
Tumour with radiotherapy	8 (9)	0 (0)	

^a^ Values are expressed as No. (%) or mean ± SD.

The causes of GH deficiency varied between the two groups. The predominant cause of GHD in the ‘GH continued’ group was pituitary surgery (53%) followed by adult-onset idiopathic isolated growth hormone deficiency (24%). On the other hand, the commonest cause of GH deficiency in the ‘GH stopped’ group was idiopathic (57%) with hypophysitis as the second commonest (28%). The differences in the distribution of the causes of GHD between the two groups was statistically significant (P < 0.001) (Table 1). 

**Figure 2.**
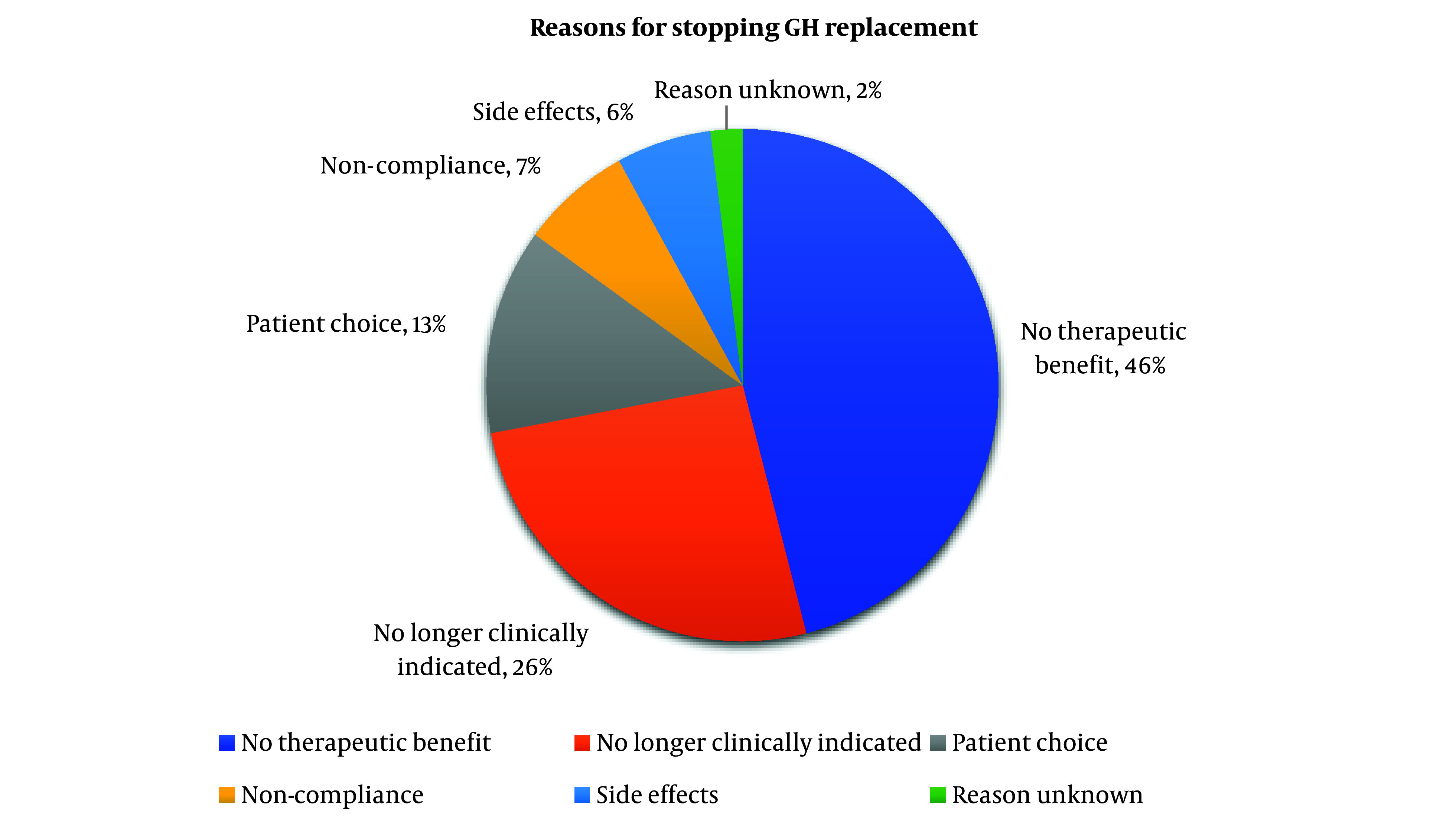
Pie chart showing reasons for stopping growth hormone among patients who discontinued growth hormone replacement.

The reasons for stopping growth hormone replacement are displayed in Figure 2. The most prevalent reason for discontinuing GH was because of ‘no perceived therapeutic benefit’, representing 25 patients (46%). This was followed by ‘no longer clinically indicated’ accounting for 14 patients (26%). Patient choice and side effects accounted for 7 (13%) and 3 (6%) patients each respectively. There was no documentation regarding the nature of the side effects, however. Notably, an instance where the reason for cessation was unknown is limited to a patient, while reported patient non-compliance was 4 cases (7%). 

From the logistics regression model, upon adjusting for AGHDA score and age, gender emerged as borderline significantly associated with the cessation of GH therapy. Females exhibited a 2.45 times higher likelihood of discontinuing GH therapy compared to males (P-value = 0.05). However, no other baseline factors displayed a statistically significant association with the cessation of GH replacement (Table 2). 

**Table 2.** Logistics Regression Model Showing Factors Associated with Cessation of Growth Hormone Replacement

Characteristic	Odds Ratio	P-Value
**AGHDA score**	0.98	0.74
**Gender (female)**	2.45	0.05
**Age**	0.98	0.18

## 5. Discussion

In this real-world study, we show that over two in five patients who stopped GH replacement did not perceive any therapeutic benefit while taking it. To the best of our knowledge, this is the first study looking at reasons why patients with severe GH deficiency stop their growth hormone replacement. Long term use of GH has been shown to improve the quality of life in patients with GHD (25, 26). The improvement in the quality of life in GH-deficient patients on replacement is perceived as a therapeutic benefit. In our study, it was not possible to tell whether the lack of perceived therapeutic benefit was correlated with low quality of life at the time of GH cessation because not all patients had QoL-AGHDA measured at the time of stopping GH. Following initiation of growth hormone replacement, there is an increase in anaerobic energy capacity. This could partly explain the improvement in quality of life as patients are able to initiate physical activity (27). The changes in body composition however have been shown to occur predominantly in the first 12 months following GH initiation (28). These changes plateau thereafter, which may be seen as a lack of therapeutic benefit after a period. In addition, these patients may also have unrealistic short-term expectations for improvement following GH initiation. It has been shown that the benefits of GH replacement in the body such as muscle strength, occurs over an extended duration of time (29). This may therefore be viewed as lack of therapeutic benefit in the short run and therefore lead to cessation of therapy. 

We have shown that compared to those who were on GH, patients who had stopped GH replacement were more likely to have an idiopathic cause of GHD. In adults, although not applicable to our patient cohort, GHD may be a continuation of childhood-onset GHD which is mostly idiopathic in nature (10). It has been shown that idiopathic childhood-onset GHD is frequently transient in nature (10, 30). The reasoning behind this is largely unknown but, it is thought to be linked to the increase in sex hormones and maturation of the GH axis. Consequently, GHD is reversed resulting in normalisation in GH secretion at a later age (31). Adult-onset idiopathic GHD on the other hand, its diagnosis may prove to be challenging due to reduced GH pulsatility and reduced peak GH response to secretagogues with increasing age (32). In addition, serum IGF-1 levels decline throughout adulthood causing an increased overlap in IGF-1 level between normal and GH-deficient adults. There are also ongoing debates about the growth hormone cut-offs following a stimulation test. The consideration of BMI to adjust for growth hormone cut-offs has been suggested (33). There is a further argument that seeks to interpret the results on a continuum starting from patients with severe GH deficiency requiring GH replacement on one end, to those with mild GH deficiency who would require monitoring, periodic testing and alternative therapies on the other end (34). It is therefore possible, we argue, that some of these patients who were initiated on GH replacement but stopped it may not have needed it in the first place. On the other hand, it is no surprise that all patients continued on replacement following pituitary surgery. Growth hormone deficiency is more likely to occur (> 95%) in patients with multiple pituitary hormone deficiencies (> 3 axes involved) after surgery. The risk of postoperative hypopituitarism depends on several factors including the operating neurosurgeon’s experience, degree of surgical manipulation and tumour size among others (35). Interestingly, a study by Jahangiri et al. described a 6-week postoperative growth hormone recovery of 22%, with a minimal change 6 months later (36). This suggests that postoperative GHD recovery can be prolonged which may necessitate GH replacement over a longer period. 

Being female increases the likelihood of stopping GH replacement by 2.5 times compared to males. This association is complex but could be related to the blunted response to GH replacement in females as compared to males as shown in previous studies. Oestrogen has been found to affect the somatotrophic axis by increasing fat mass and reducing IGF-1 synthesis in the liver due to its first-pass metabolism effect. This is especially observed in orally administered oestrogen in postmenopausal women as it subjects the liver to first-pass metabolism of its supraphysiological doses (37-40). Testosterone on the other hand, enhances the effects of GH replacement in men by increasing muscle mass and IGF-1 levels. As a result, GH replacement produces a difference in body composition and IGF-1 levels between the two genders (41-44). In addition, a higher dose of GH replacement is required in females as compared to males to achieve similar positive effects (44, 45). In addition, a longer duration may also be taken to establish a maintenance GH dose in women (46). This diminished IGF-1 responsivity to GH replacement in GH-deficient females may be perceived as a lack of therapeutic benefit compared to men, leading to GH treatment cessation.

Our study has several limitations. We did not collect data on potential confounders which may affect the prediction for GH cessation. Data on comorbidities, AGHDA score at GH cessation, concomitant medication including other hormonal replacements may have an impact on the logistic regression model. We have however shown that patients with GHD due to pituitary surgery (which most times leads to partial or panhypopituitarism with resultant multiple hormone replacement) are likely to keep on GH replacement. We also excluded patients with no cause of GH deficiency and no GH start or stop date (Figure 1). This could have introduced a bias in the sample chosen for analysis. Further still, we have not explored genetic causes of GHD in adults which could be a continuation of childhood GHD and likely to be permanent. Nonetheless, we have shown a distribution in the causes of GHD which is representative of a typical tertiary centre cohort. 

In conclusion, this real-world investigation of patients with severe GH deficiency has shown that over two in five individuals who discontinued GH therapy cited the absence of perceived benefits. Notably, gender is a predictive factor for the cessation of GH replacement, with females displaying a higher tendency to discontinue this therapy. Further research into the long-term effects of stopping GH replacement is needed to provide more understanding to help physicians tailor GH treatment more effectively.

## Data Availability

The dataset presented in the study is available on request from the corresponding author during submission or after its publication. The data are not publicly available due to privacy.
